# Evaluation of the Relationship Between Diabetic Retinopathy and the Development of Left Atrial Stiffness in Patients with Diabetes Mellitus

**DOI:** 10.5152/eurasianjmed.2023.23235

**Published:** 2023-10-01

**Authors:** Oğuzhan Birdal, Mehmet Saygı, Remziye Doğan, Levent Pay, Emrah Aksakal, Caner Topaloğlu, Mustafa Yıldırım, Uğur Aksu

**Affiliations:** 1 Department of Cardiology, Atatürk University Faculty of Medicine, Erzurum, Türkiye; 2 Department of Cardiology, Hisar Intercontinental Hospital, İstanbul, Türkiye; 3 Department of Cardiology, Ardahan State Hospital, Ardahan, Türkiye; 4 Department of Cardiology, Erzurum City Hospital, Erzurum, Türkiye; 5 Department of Cardiology, İzmir University of Economics, İzmir, Türkiye; 6 Department of Ophthalmology, Medical Faculty, Atatürk Üniversitesi, Erzurum, Türkiye; 7 Department of Cardiology, Afyonkarahisar Health Sciences University, Afyon, Türkiye

**Keywords:** Diabetes mellitus, LA stiffness, MAPSE, retinopathy

## Abstract

**Objective:**

Based on several studies, atrial remodeling results in an increase in left atrial (LA) stiffness, which is indicative of a worsened reservoir function. A typical microvascular consequence of diabetes mellitus (DM) is diabetic retinopathy. Therefore, the objective of this study was to assess the factors that might be related to LA stiffness in DM patients.

**Materials and Methods:**

There were 200 DM patients in the study population. The LA stiffness value of 0.33 led to the division of the patients into 2 groups. According to these groups, the parameters to predict the development of the LA stiffness were investigated.

**Results:**

The patient population’s median age was 54.7 ± 9.4 years, and of them, 105 (52.5% of the population) were men. Retinopathy was substantially linked with LA stiffness. Interventricular septum thickness (B coefficient: 0.261, 95% CI 0.128; 0.394; *P* < .001), mitral annular plane systolic excursion (B coefficient: −0.350, 95% CI −0.489; −0.2212; *P* < 0.001), and retinopathy (B coefficient: 0.644, 95% CI 0.307; 0.983; *P* < .001) were identified as independent predictors of the progression of LA stiffness by the linear regression model.

**Conclusion:**

The results of the current investigation demonstrated a correlation between higher LA stiffness values and the presence of diabetic retinopathy in diabetic patients.

**Main Points**

- Left atrial (LA) stiffness is an important parameter used to determine LA functions.
- Diabetic retinopathy is an independent predictor of the LA stiffness development.
- A significant relationship was found between LA stiffness and mitral annular plane systolic excursion and interventricular septum.

## Introduction

Diabetes mellitus (DM) is a chronic, multisystemic, and metabolic disease that requires constant monitoring for complications.^1^ A typical and distinct microvascular consequence of DM is diabetic retinopathy. It is also thought to be the main reason for avoidable blindness in people aged 20 to 74 in developed countries.^2^

Independent of cardiovascular risk factors, retinopathy has been linked to a higher risk of stroke, coronary heart disease, and heart failure.^3-5^ These results show that diabetic retinopathy is a characteristic of diffuse end-organ microcirculatory damage and that patients with diabetic retinopathy need to be closely monitored for cardiovascular events.^6^

The stepwise backward effects of loss in left atrial (LA) functional properties are a reduction in lung vessel compliance and vascular remodeling that result in right ventricular overload and dysfunction. The left atrium is extremely sensitive to sustained volume and pressure overload secondary to increased left ventricular filling pressures. The volumetric and longitudinal deformation indices of the left atrium have a significant linear connection, in contrast to left ventricular measurements. Evaluation of many cardiac disorders requires early identification of subclinical LA dysfunction.^7^ A relatively novel parameter that reflects LA functions is LA stiffness. Patients without symptoms of cardiovascular illness have previously studied the impact of type 2 DM on LA remodeling.^8^ In this study, patients with diabetic retinopathy were assessed in terms of LA stiffness and their association, suggesting that disorders that impact LA structure and compliance merit further research.

## Materials and Methods

### Data Collection

Before any data were collected, the study was approved by the Atatürk University Faculty of Medicine and all participants provided informed consent (Date: July 6, 2023; Approval no: 5, 87-582) undertaken in accordance with the Helsinki Declaration. Two hundred DM patients who applied to our outpatient clinic between January 2019 and April 2022 make up the study population. The hospital database was used to gather demographic information about the patients as well as clinical results. Using the most recent American Diabetes Association diagnostic standards, DM was identified.^9^ Patients under the age of 18, those with retinopathy from another cause, and those whose echocardiographic data could not be correctly assessed were eliminated from the study. An ophthalmologist made the diagnosis of diabetic retinopathy based on the clinical appearance of retinal vascular anomalies.^10,11^ A peripheral vein was used to draw blood for hematological and biochemical analyses.

Echocardiography analysis: A cardiovascular imaging specialist used the Vivid 7 (GE Vingmed Ultrasound AS, Horten, Norway) device to perform transthoracic echocardiography on each patient. The modified Simpson’s method was used to calculate the left ventricular ejection fraction (LVEF), and apical 2- and 4-chamber images were used to assess the left ventricular end-diastolic and end-systolic volumes. The evaluation of LV filling models, mitral septal annular velocity, and pulse wave velocity followed the most recent recommendations from the American Society of Echocardiography.^12^ The LA strain was determined by averaging the LA strain for each patient’s 2-chamber and 4-chamber views. The LA reservoir strain corresponds to the first peak LA strain that was recorded at the conclusion of systole. Early mitral inflow velocity to early annular tissue velocity (E/e) was used to construct the LA stiffness index, which was then compared to the LA reservoir strain.^13^

### Statistical Analysis

The statistical analyses were carried out using the software package Statistical Package for the Social Sciences, version 26.0 (IBM SPSS Corp.; Armonk, NY, USA). The G Power Package program was used to do a power study and determine the patient count. The median LA stiffness value was used to split the patients into 2 groups. Group 1 patients had a median LA stiffness of less than or equal to 0.33, whereas group 2 patients had a median LA stiffness of more than 0.33. The descriptive statistics derived from the collected data were expressed as mean standard deviation or median (interquartile range) values when continuous variables were found to either conform or not conform to the normal distribution, and when categorical variables were involved, they were expressed as percentage values. Baseline characteristics were classified and evaluated using appropriate statistical tests, such as the independent samples *t*-test for normally distributed continuous variables, the Mann–Whitney *U*-test for non-normally distributed continuous variables, and the appropriate chi-square test for categorical variables, based on predetermined subgroups. The linear regression analysis took into account variables that were statistically significant in the univariate analysis. High-correlation parameters were excluded from the regression analysis. Probability (*P*) statistics of .05 or less were regarded as indicating statistical significance.

## Results

**Figure 1.**
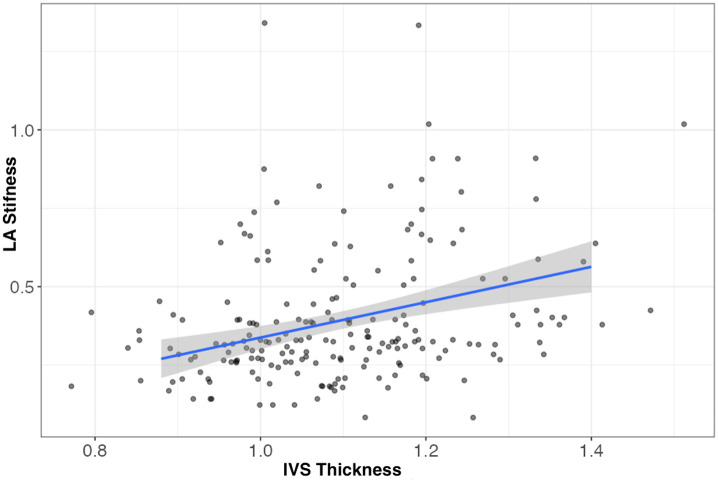
Correlation of left atrial stiffness and interventricular septum thickness. IVS, interventricular septum; LA, left atrial.

**Figure 2.**
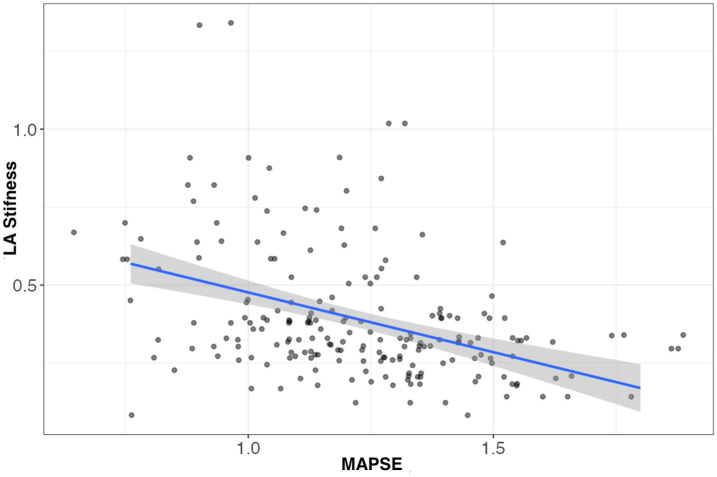
Correlation of left atrial stiffness with mitral annular plane systolic excursion. LA, left atrial; MAPSE, mitral annular plane systolic excursion.

**Figure 3.**
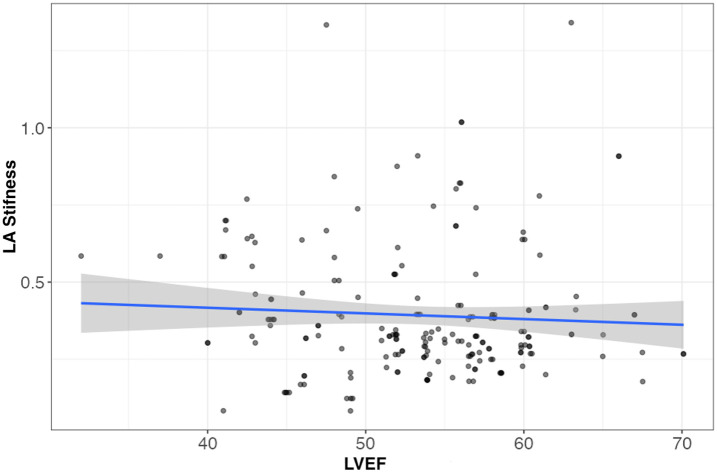
Correlation of left atrial stiffness with left ventricular ejection fraction. LA, left atrial; LVEF, left ventricle ejection fraction.

Two hundred patients were included in the study according to the inclusion and exclusion criteria. Hundred patients with LA stiffness values 0.33 were included in group 1, and 100 patients with values > 0.33 were included in group 2. The mean age of the patients was 54.7 ± 9.4 years, and 105 (52.5%) of the patients were male. Age and gender did not significantly differ between groups 1 and 2 (*P* = .051 and *P* = .852, respectively). Univariate analysis revealed a statistically significant relationship between the groups in terms of height (group 2 vs. group 1; 173.3 ± 6.1 vs. 168.1 ± 6.2, *P* < .001), albumin (group 2 vs. group 1; 4.4 ± 0.4 vs. 4.1 ± 0. 3, *P* = .003), total cholesterol (group 2 vs. group 1; 214.4 ± 53.6 vs. 193.3 ± 45.5, *P* = .005), and retinopathy (group 2 vs. group 1; 69 (69%) vs. 50 (50%), *P* = .006) rates. Baseline demographic and clinical characteristics are shown in Table 1. In echocardiographic data, there was a statistically significant relationship between the groups in interventricular septum (IVS) thickness; posterior wall (PW) thickness; mitral annular plane systolic excursion (MAPSE); LV septal Sm (peak myocardial velocity during systole), LV septal Em (peak myocardial velocity during early systole), LV septal Am (peak myocardial velocity during atrial contraction); LV global longitudinal strain; and left atrial volume index values (*P* = .034; *P* < .001; *P* = .043; *P* = .032, *P* < .001, *P* = .003; *P* = .021; *P* = .033, respectively). Echocardiographic parameters are detailed in Table 2. Significant relationships between LA stiffness and IVS and LA stiffness with MAPSE are shown in Figures 1 and 2, but the same relationship could not be shown between LA stiffness and LVEF (Figure 3). In the linear regression analysis to find the predictors of LA stiffness, the parameters that were significant in the univariate analysis were included in the linear regression model and the predictors of stiffness were investigated. In this model, IVS thickness (B coefficient: 0.261, 95% CI 0.128; 0.394; *P* < .001), MAPSE (B coefficient: −0.350, 95% CI −0.489; −0.2212; *P* < .001), and retinopathy (B coefficient: 0.644, 95% CI 0.307; 0.983; *P* < .001) were found to be independent predictors of the development of LA stiffness.

## Discussion

The results of the present investigation showed that the development of LA stiffness is independently predicted by diabetic retinopathy. Furthermore, it was discovered that MAPSE and IVS were independent predictors of LA stiffness prediction.

Using PW (E) and tissue Doppler (é) measurements that show LV diastolic functions and LA strain values, LA stiffness is a crucial metric used to predict LA function, notably compliance.^14^ Pilote et al^15^ originally described stiff LA syndrome in 1988, 7 years following mitral valve surgery, when substantial pulmonary hypertension was found despite the prosthetic mitral valve’s absence of malfunction. It has been described as a phenomenon that happens years after cardiac surgery and results in the development of pulmonary hypertension for no other reason. Following catheter ablation for atrial fibrillation (AF), it was discovered that individuals, particularly those with a history of numerous ablations, could develop stiff LA syndrome and the associated pulmonary hypertension, and that a high LA stiffness could be a significant factor in predicting AF recurrence.^16,17^

It is crucial to understand the variables that predict LA stiffness because it is linked to significant cardiovascular events. It is understood that catheter ablation and valve surgery alter the left atrium’s architectural and physiological structure and result in an increase in LA stiffness.^15,16^ In addition to these, it is critical to highlight the diseases linked to LA stiffness, which reduce the left atrium’s compliance by leading to scarring and fibrosis. Endothelial dysfunction, subclinical atherosclerosis, and elevated low-density lipoprotein values were discovered to be connected to LA dysfunction in a study done on hypertensive patients.^18^

Previous studies have looked into the connection between DM, LA, and AF function. Kim et al^19^ demonstrated that in patients having catheter ablation for AF, DM was linked to increased LA stiffness. According to Kaze et al,^20^ diabetic individuals have a higher chance of developing AF due to DM-related microvascular problems like diabetic nephropathy, neuropathy, and retinopathy. One of the key signs of microvascular impairment in DM patients is diabetic retinopathy.^21^ Although there are studies demonstrating that diabetic retinopathy is linked to the onset of AF, to the best of our knowledge, this study is the first to examine diabetic retinopathy as a predictor of LA stiffness.

Recent research has also revealed that LA stiffness is much higher in individuals with paroxysmal and chronic AF episodes as well as in AF patients undergoing catheter ablation.^22^ Left atrial stiffness may be a significant predictor of AF development because it was higher in patients with persistent AF compared to those with paroxysmal AF.^23^ High LA stiffness was also found to be a significant predictor of early AF recurrence by Marino et al.^24^ In heart failure with reduced ejection fraction and preserved ejection fraction other than AF, it has been demonstrated that LA stiffness also affects death, hospitalization, effort capacity, and echocardiographic abnormalities, and high LA stiffness is linked to negative outcomes.^14,25^

In our study, we have identified both MAPSE and IVS, in addition to diabetic retinopathy, as independent predictors in foreseeing LA stiffness. As a well-established echocardiographic parameter, MAPSE has been recognized in prior studies as an early indicator of left ventricular dysfunction and myocardial fibrosis. Furthermore, it holds prognostic significance in conditions such as atrial fibrillation, heart failure, and post-myocardial infarction cases.^26^ A recent study has also pinpointed MAPSE as a noteworthy predictor for LA stiffness.^27^ The augmentation of IVS thickness has long been acknowledged as a characteristic feature, particularly indicative of diastolic dysfunction in the heart. A recent study has unveiled a correlation between the escalation in IVS thickness among hypertensive patients and subclinical LA dysfunction.^28^

Our study’s main drawback is that it was conducted retrospectively at a single site with a small number of patients. Additionally, we were unable to assess the severity of diabetic retinopathy or if it had any effect on LA stiffness due to the dearth of data in our study. Prospective studies on this topic including more patients are required to determine whether the severity of diabetic retinopathy affects its effectiveness.

In conclusion, we discovered that high LA stiffness values were related to the occurrence of diabetic retinopathy in diabetic patients. Therefore, in diabetic patients with diabetic retinopathy, it would be appropriate to be more cautious regarding LA stiffness and associated cardiovascular diseases.

## Figures and Tables

**Table 1.** Baseline Demographic Clinic Characteristics Comparison According to Group 1 and Group 2

Variables	Low LA Stiffness (n : 100)	High LA Stiffness (n : 100)	*P*
Age (year)	53.6 ± 10.2	55.9 ± 8.6	.051
Gender, male (n, %)	54 (54)	51 (51)	.852
Systolic blood pressure (mmHg)	135.2 ± 18.1	139.1 ± 19.7	.145
Diastolic blood pressure (mmHg)	79.9 ± 11.5	83 ± 12.1	.055
Heart rate (bpm)	85.3 ± 11.5	85.9 ± 13.7	.712
Pulse pressure (mmHg)	56.7 ± 15.3	59.1 ± 19.3	.327
Height (cm)	168.1 ± 6.2	173.3 ± 6.1	**<.001**
Weight (kg)	78.7 ± 12.6	76.5 ± 13.7	.242
BMI	29.6 ± 4.7	30.2 ± 5.4	.422
Statin usage (n, %)	68 (68)	79 (79)	.078
Hypertension (n, %)	36 (36)	32 (32)	.550
Smoking (n, %)	26 (26)	21 (%21)	.389
Glucose (mg/dL)	204.4 ± 82.1	220.6 ± 105.9	.259
Creatinine (mg/dL)	0.9 ± 0.3	0.8 ± 0.3	.527
Albumin (mg/dL)	4.1 ± 0.3	4.4 ± 0.4	**.003**
Total cholesterol (mg/dL)	193.3 ± 45.5	214.4 ± 53.6	**.005**
TSH (µIU/mL)	1.3 ± 0.6	2.5 ± 0.7	.113
HbA1c (%)	8.2 ± 1.6	8.6 ± 2	.180
Microalbuminuria (mg/day)	67.8 ± 161	65.5 ± 149	.921
WBC (10^9^/L)	7.7 ± 2	7.6 ± 1.5	.742
Hb (g/dL)	13 ± 1.5	12.6 ± 12.8	.059
Retinopathy (n, %)	50 (50)	69 (69)	**.006**

BMI, body mass index; Hb, hemoglobin; HbA1c, glycated hemoglobin; LA, left atrial; TSH, thyroid-stimulating hormone; WBC, white blood cells.

**Table 2.** Echocardiographic Findings

Variables	Low LA Stiffness (n : 100)	High LA Stiffness (n : 100)	*P*
IVS thickness	1.1 ± 0.1	1.3 ± 0.1	**.034**
PW thickness	1 ± 0.1	1.2 ± 0.1	**<.001**
LVEDD	4.7 ± 0.5	4.8 ± 0.3	.949
LVESD	30 ± 0.4	2.9 ± 0.5	.280
LVEF	53.3 ± 7	53.5 ± 6.9	.839
TAPSE	2.3 ± 0.3	2.2 ± 0.3	.578
MAPSE	14 ± 0.3	12 ± 0.2	**.043**
LV septal Sm	0.175	0.115	**.032**
LV septal Em	0.195	0.07	**<.001**
LV septal Am	0.180	0.09	**.003**
LV GLS	18.7 ± 2.4	17.9 ± 2.5	**.021**
LAVI	42.2 ± 17.4	47.4 ± 13.1	**.033**
LA stiffness	0.3 ± 0.1	0.5 ± 0.2	**<.001**
LA reservoir strain	30.1 ± 6.1	23.4 ± 5.9	**<.001**
LA conduit strain	13.8 ± 3.9	12.8 ± 3.9	.090
LA contractile strain	16.4 ± 6.4	10.6 ± 6.4	**<.001**
LA strain rate Sm	1.3 ± 0.5	1.2 ± 0.3	.065
LA strain Em	1.2 ± 0.6	0.8 ± 0.5	**<.001**
LA strain Am	1.7 ± 0.6	1.6 ± 0.6	**.045**

Am, peak myocardial velocity during atrial contraction; Em, peak myocardial velocity during early systole; GLS, global longitudinal strain; IVS, interventricular septum; LA, left atrial; LAVI, left atrial volume index; LVEDD, left ventricle end-diastolic diameter; LVEF, left ventricular ejection fraction; LVESD, left ventricle end-systolic diameter; MAPSE, mitral annular plane systolic excursion; PW, posterior wall; Sm, peak myocardial velocity during systole; TAPSE, tricuspid annular plane systolic excursion.

**Table 3.** Left atrial stiffness predictors

Variables	β-Coefficient	CI (95%)	*P*
Age	−0.004	−0.157-0.149	.96
Height	−0.141	−0.302-0.019	.08
Diastolic blood pressure (mmHg)	−0.014	−0.156-0.127	.84
Albumin	−0.053	−0.202-0.095	.48
Total cholesterol	0.12	−0.027-0.267	.11
Hemoglobin	0.016	−0.151-0.184	.84
IVS thickness	0.261	0.128-0.394	**<.001**
LV GLS	−0.012	−0.177-0.153	.88
LAVI	0.093	−0.058-0.242	.22
MAPSE	−0.350	−0.489-0.2212	**<.001**
Retinopathy	0.644	0.307-0.983	**<.001**

GLS, global longitudinal strain; IVS, interventricular septum; LAVI, left atrial volume index; LV, left ventricle; MAPSE, mitral annular plane systolic excursion.
